# A Survey of 3D Reconstruction: The Evolution from Multi-View Geometry to NeRF and 3DGS

**DOI:** 10.3390/s25185748

**Published:** 2025-09-15

**Authors:** Shuai Liu, Mengmeng Yang, Tingyan Xing, Ran Yang

**Affiliations:** 1 School of Artificial Intelligence, China University of Geosciences (Beijing), Beijing 100083, China; 2104230092@email.cugb.edu.cn; 2 School of Vehicle and Mobility, Tsinghua University, Beijing 100084, China; yangmm_qh@tsinghua.edu.cn; 3 College of Resource Environment and Tourism, Capital Normal University, Beijing 100048, China; 2220901014@cnu.edu.cn

**Keywords:** 3D reconstruction, deep learning, multiple view geometry, NeRF, 3DGS

## Abstract

Three-dimensional (3D) reconstruction technology is not only a core and key technology in computer vision and graphics, but also a key force driving the flourishing development of many cutting-edge applications such as virtual reality (VR), augmented reality (AR), autonomous driving, and digital earth. With the rise in novel view synthesis technologies such as Neural Radiation Field (NeRF) and 3D Gaussian Splatting (3DGS), 3D reconstruction is facing unprecedented development opportunities. This article introduces the basic principles of traditional 3D reconstruction methods, including Structure from Motion (SfM) and Multi View Stereo (MVS) techniques, and analyzes the limitations of these methods in dealing with complex scenes and dynamic environments. Focusing on implicit 3D scene reconstruction techniques related to NeRF, this paper explores the advantages and challenges of using deep neural networks to learn and generate high-quality 3D scene rendering from limited perspectives. Based on the principles and characteristics of 3DGS-related technologies that have emerged in recent years, the latest progress and innovations in rendering quality, rendering efficiency, sparse view input support, and dynamic 3D reconstruction are analyzed. Finally, the main challenges and opportunities faced by current 3D reconstruction technology and novel view synthesis technology were discussed in depth, and possible technological breakthroughs and development directions in the future were discussed. This article aims to provide a comprehensive perspective for researchers in 3D reconstruction technology in fields such as digital twins and smart cities, while opening up new ideas and paths for future technological innovation and widespread application.

## 1. Introduction

3D reconstruction technology, as one of the pivotal technologies in the realms of computer vision and graphics, is dedicated to the task of transforming real-world objects and scenes into intricate digital 3D models. With the rapid advancements in various sectors, including VR/AR, autonomous driving, smart cities, and cultural heritage preservation, the application of 3D reconstruction technology has witnessed a significant broadening of its scope and has emerged as a pivotal force in driving digital transformation and intelligent development. Three-dimensional reconstruction can accurately replicate the complex structures and dynamic changes in the real world by utilizing high-precision modeling and detailed scene restoration, providing strong support for many fields such as urban planning, infrastructure construction, and cultural relic protection [1].

Traditional 3D reconstruction methods have yielded significant results in static scenes; their limitations in terms of computational efficiency, accuracy, and adaptability have become increasingly prominent as the demand for dynamic environments and complex scenes grows. For instance, when reconstructing large-scale and intricate scenes, traditional methods often encounter challenges such as high computational complexity and difficulties in data collection [2]. In response to these challenges, recent years have witnessed significant advancements in the field of 3D reconstruction, driven by innovations in deep learning technology and novel view synthesis. For example, Neural Radiation Field (NeRF) integrates implicit representations with deep neural networks to achieve high-quality scene rendering from various perspectives, presenting more realistic details and lighting effects [3]. On the other hand, 3D Gaussian Splatting (3DGS) utilizes explicit Gaussian primitives to represent scenes and performs novel view synthesis, thereby enhancing rendering quality (i.e., the visual fidelity and detail of the rendered images) and efficiency, and expanding the potential applications of 3D reconstruction technology in dynamic scenes [4]. Although these emerging technologies have made breakthroughs in certain fields, 3D reconstruction technology still faces numerous challenges in real-time rendering, dynamic scene processing, and efficient data handling. Therefore, future research must not only tackle these technological bottlenecks but also further integrate deep learning with traditional geometric methods to enhance the adaptability and practical effectiveness of 3D reconstruction technology in complex environments [5].

In this context, in recent years, several review articles have systematically summarized 3D reconstruction, but their research perspectives differ. Some reviews offer a comprehensive overview of traditional geometry-based 3D reconstruction techniques [1], primarily focusing on the principles, workflows, and optimization strategies of Structure from Motion (SfM) and Multi-View Stereo (MVS). These works emphasize geometric accuracy, reconstruction stability, and applicability to static scenes; however, they provide limited coverage of deep learning methods and implicit representation techniques, and lack discussion on reconstruction in complex and dynamic scenarios. Other studies focus on implicit representation methods based on Neural Radiance Fields (NeRF), systematically reviewing NeRF’s theoretical foundations, network design, and multi-view rendering quality [6,7]. They highlight its advantages in detail recovery and lighting handling, while also pointing out issues such as high training costs, sensitivity to sparse view data, and limited adaptability to dynamic scenes. Nevertheless, these works primarily concentrate on implicit representation methods themselves, with little comparison to traditional SfM/MVS or emerging explicit methods such as 3D Gaussian Splatting (3DGS), and thus lack cross-generational technical analysis. Additionally, some reviews focus on the overall development of deep learning-based 3D reconstruction methods [8], including convolutional neural networks, graph neural networks, and self-supervised learning, systematically summarizing model design and training strategies, but providing relatively limited discussion on rendering quality and computational efficiency. Other studies concentrate on recent advances in 3DGS [9], analyzing Gaussian primitive representations, sparse view synthesis, and rendering efficiency optimization. However, these works do not sufficiently position 3DGS relative to traditional geometry-based methods and NeRF approaches, nor do they provide in-depth analysis for dynamic or large-scale scene reconstruction.

In summary, although existing review articles possess a certain degree of systematicity and depth within their respective focuses, they still exhibit notable limitations. These are mainly reflected in the fact that most reviews concentrate on a single category of methods, lacking cross-generational comparisons and thus failing to comprehensively present the technical evolution and relationships among traditional geometry-based methods, implicit representation methods, and emerging explicit approaches. In addition, multi-dimensional analyses of key metrics such as rendering quality, computational efficiency, sparse-view support, and adaptability to dynamic scenes remain insufficient. Moreover, discussions on future development trends and potential research challenges are relatively limited.

To address these gaps, this paper proposes a cross-generational and multi-dimensional review perspective, with its novelty highlighted in three aspects:

**(1)**   Systematic review of technological evolution: This article starts from the traditional Structure from Motion (SfM) [10,11,12,13,14,15,16] and Multi View Stereo (MVS) [17,18,19,20,21] methods, this paper details their development history and further summarizes emerging methods based on Neural Radiance Fields (NeRF) and 3D Gaussian Splatting (3DGS), presenting the cross-generational evolution of 3D reconstruction techniques from explicit geometric modeling to implicit depth representation.
**(2)**   Multi-dimensional comparative analysis: From key dimensions including rendering quality, computational efficiency, sparse-view support capability, and dynamic scene reconstruction, this paper conducts a comprehensive comparison of different methods, revealing their respective strengths and limitations, thereby providing researchers with guidance for understanding technical characteristics and selecting appropriate approaches.
**(3)**   Identification of challenges and future directions: Based on the systematic review and comparative analysis, this paper summarizes the main challenges in real-time rendering, dynamic scene processing, and efficient data management in 3D reconstruction, and explores potential research breakthroughs and future development directions, aiming to provide systematic guidance for subsequent studies and to promote technological progress and practical application of the field.

**Figure 1:**
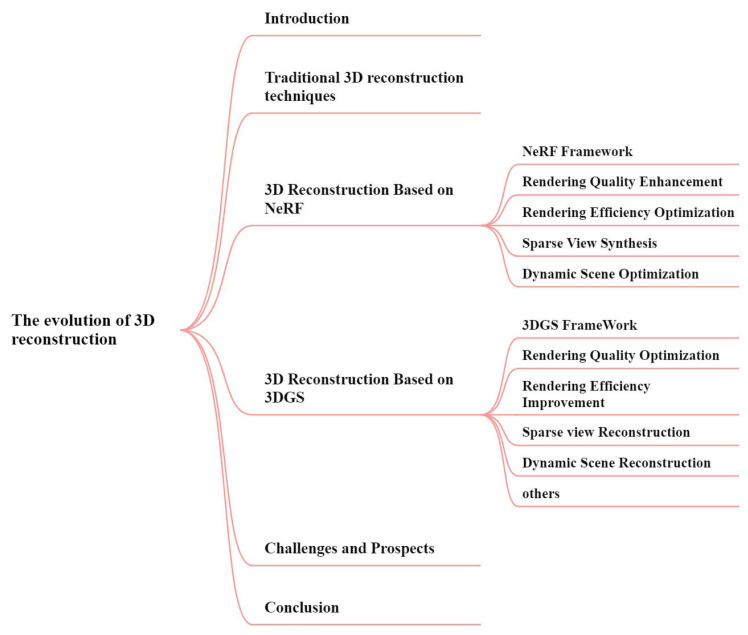
Framework of the Literature Review.

The overall structure of this article is illustrated in Figure 1.

## 2. Traditional 3D Reconstruction Techniques

**Figure 2:**
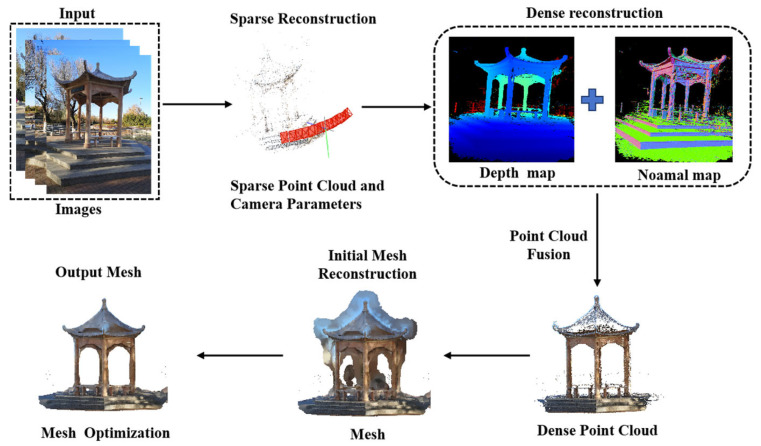
Three-dimensional Reconstruction Process Based on Multi-View Geometry.

The traditional 3D reconstruction method based on multi view geometry theory has significant advantages such as intuitive visualization, clear structural expression, and strong compatibility. Classic algorithms, such as SfM, generate sparse point clouds and accurately estimate the camera pose by matching feature points between images and applying geometric principles. Building upon this, MVS technology, based on multi-view geometry, further computes dense point clouds through dense matching of multi-view images and employs advanced algorithms such as Poisson surface reconstruction [22] or Delaunay triangulation [23,24] to generate precise mesh model. Within this multi-view geometry framework, commonly used feature matching algorithms include Scale-Invariant Feature Transform (SIFT) [25], Speeded-Up Robust Features (SURF) [26] and Oriented FAST and Rotated BRIEF (ORB) [27]. These methods effectively extract stable feature points from images, providing a reliable foundation for 3D reconstruction. However, early 3D reconstruction algorithms typically relied on pre-calibrated cameras or motion along specific trajectories, which limited their applicability and allowed effective reconstruction only in static and known environments. The 3D reconstruction process based on multi-view geometry is shown in Figure 2.

With the continuous development of 3D reconstruction technology, especially between the 1990s and 2000s, the hierarchical reconstruction theory based on projective geometry significantly enhanced the robustness of algorithms. Through a computational framework that progresses from projective space to affine space and then to Euclidean space, this theory effectively reduces the number of unknown variables, giving the reconstruction process a clearer geometric meaning. This breakthrough laid a solid theoretical foundation for the further development of modern 3D reconstruction technologies. In recent years, with further advancements in technology, many innovative achievements have emerged in the field of 3D reconstruction. For example, Hartley et al. [28] conducted in-depth research on multi-view geometry theory, and the algorithms they proposed have been widely applied in computer vision and robotics. Moons et al. [29] proposed a method for object surface shape estimation and viewpoint parameter solving based on multiple images, further enriching the multi-view geometry theory system. In addition, [30] introduced a novel multi-view stereo algorithm that intelligently selects images for matching, providing an effective solution for efficiently processing 3D reconstruction from large collections of community photos. Li et al. [31] proposed a new method for modeling and recognizing landmark image sets based on iconic scene graphs, opening up new directions in the field of landmark recognition. In the context of large-scale 3D reconstruction applications. Zhu et al. [32] presented a global SfM method based on distributed motion averaging. By constructing a distributed processing framework and incorporating data fusion techniques, they significantly improved the efficiency and accuracy of 3D reconstruction, making it particularly suitable for processing ultra-large-scale datasets. With the continuous development of deep learning technology, more and more studies are dedicated to applying it to traditional 3D reconstruction methods to improve the accuracy of feature extraction and matching, as well as overall reconstruction efficiency. Among them, MVSNet [33] first proposed an end-to-end deep learning MVS framework that can directly regress dense depth maps from unordered multi view images, thereby restoring the 3D structure of the scene. Fast-mvsnet [34] proposes a sparse-to-dense MVS framework that utilizes lightweight networks to estimate initial sparse depth points from image pairs, avoiding the need to construct a complete cost volume and significantly reducing computational complexity. By combining learning guided deep propagation with Gauss–Newton optimization, the efficiency and stability of reconstruction are improved. Compared to the static and fixed depth sampling strategy of MVSNet, Wang et al. [35] introduces an iterative dynamic cost volume mechanism, which dynamically adjusts the sampling range based on the current depth estimation in each iteration, effectively reducing computational overhead and improving reconstruction accuracy.

The traditional 3D reconstruction methods have developed very maturely so far, especially the oblique photography mesh model [36] which has been widely used in mainstream technologies such as digital twins, urban modeling, and GIS systems over the past decade. However, with the increasing demand for real-time rendering, dynamic scene support, and refined expression, traditional 3D modeling has gradually exposed obvious limitations. Specifically, it includes: (1) low efficiency: relying on a large number of high overlap images and complex spatial encryption processes, long generation cycles, high hardware barriers, and difficulty in efficiently modeling large-scale scenes; (2) High editing and maintenance costs: Model modifications require manual manipulation of vertices or re mapping, which is cumbersome and inefficient; (3). Weak ability to express complex structures and weak texture areas: difficult to restore complex or texture missing areas such as power poles, stairs, glass, etc., which can easily result in artifacts or loss of details; (4) Poor adaptability to lighting: The texture of the model is highly dependent on the lighting conditions during shooting, which can lead to inconsistent brightness in different lighting environments, requiring additional adjustment of material parameters; (5) Insufficient dynamic changes: The generated model is static, and when facing dynamic objects or scene updates (such as pedestrians, vehicles, and construction), data needs to be collected again, which is difficult to meet real-time requirements.

## 3. Three-Dimensional Reconstruction Based on NeRF

With the development of deep learning and neural networks, NeRF [3] has received increasing attention in the field of 3D reconstruction due to its high fidelity reconstruction capability and spatial continuity expression. It implicitly expresses the geometric and lighting characteristics of the scene through neural networks, synthesizes images from new perspectives through volume rendering, without the need for explicit generation of 3D point clouds or mesh construction. Compared with traditional methods, rendering models have more realistic lighting effects and fine textures, which are particularly prominent in complex scene reconstruction. The continuous function’s representation of the scene enables it to maintain the continuity of the scene when rendering from any perspective, avoiding the perspective jumps of traditional methods.

### 3.1. NeRF Framework

**Figure 3:**
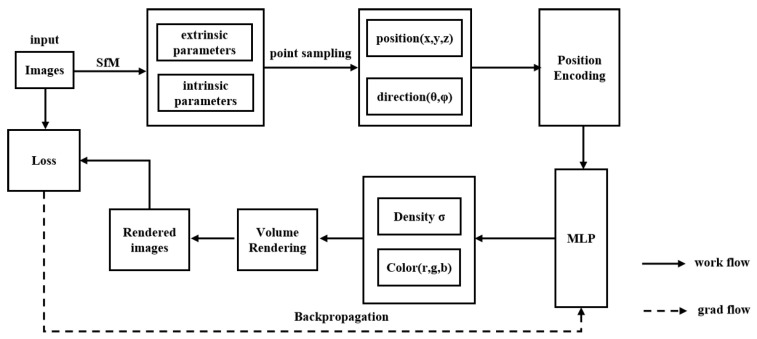
Overall Workflow of NeRF, adapted from [3].

NeRF relies on two-dimensional (2D) images for supervision, models the scene as a continuous 5D function (spatial coordinates x, y, z and view directions θ, ϕ) using a Multi-Layer Perceptron (MLP) network, and synthesizes images from novel viewpoints using volume rendering techniques. It avoids the use of traditional complex physical models and can realistically reproduce intricate 3D scenes. The pipeline is illustrated in Figure 3.

The volume rendering process used by Mildenhall et al. [3] is as follows:

(1) $$\hat{C}\left(r\right)=\sum_{i=1}^{N}T_{i}(1-exp(-\sigma_{i}\delta_{i}))c_{i},where\;T_{i}=exp(-\sum_{j=1}^{i-1}\sigma_{i}\delta_{i})$$

Among them, *N* is the number of sampling points on the same ray, $c_{i}$ is the color of the current sampling point, $\sigma_{i}$ is the opacity, $\delta_{i}$ is the distance between adjacent sampling points. $T_{i}$ is the transmittance from the current sampling point to the camera.

In recent years, research on NeRF has gradually focused on several key areas, including improving rendering efficiency [37], optimizing few-view synthesis [38,39], enhancing rendering quality, and optimizing dynamic scenes [40]. These studies are primarily dedicated to improving the performance of NeRF in real-time rendering, reducing computational overhead, and increasing the accuracy and generalization capability of 3D reconstruction, especially in cases with limited data acquisition and fewer viewpoints, while still being able to efficiently generate high-quality rendered images. Additionally, through exploration of dynamic scene modeling, NeRF not only handles the reconstruction of static objects but also adapts to complex temporal changes and object motion, thus expanding its potential in dynamic applications such as video generation, VR/AR.

### 3.2. Rendering Quality Enhancement

Rendering quality is the core key to 3D reconstruction, and improving rendering quality for NeRF has become a focus of research. In recent years, extensive efforts have been made to improve the rendering quality of NeRF, and many innovative methods have been proposed. These studies not only aim to enhance the realism and details of rendered images but also explore several key dimensions, including optimizing the model training process, improving network architecture, refining feature representation, and enhancing lighting models to address issues such as uneven lighting, aliasing, artifacts, occlusion, and motion blur in scenes. Self-supervised learning and multi view consistency strategies have been incorporated to reduce reliance on large amounts of annotated data while improving generalization ability in complex scenarios.

**Figure 4:**
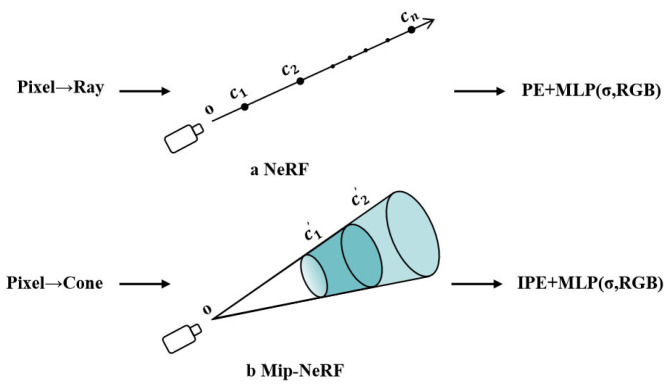
Comparison between NeRF and Mip-NeRF, $c_{i}$ is the sampling point and $c_{i}^{\prime }$ is the conical frustum, adapted from [41].

To address the aliasing and distortion issues in high-frequency details, Mip-NeRF [41] incorporates a novel Integrated Positional Encoding (IPE) and replaces the single ray in NeRF with a cone (or frustum), thereby incorporating additional domain information during the sampling process. This innovation effectively mitigates the aliasing problem caused by insufficient sampling in traditional NeRF, while preserving fine rendering details. However, Mip-NeRF does not fully account for factors such as lighting variations and image blurring, and its computational requirements are significantly higher, resulting in a notable increase in training time. A comparison between NeRF and Mip-NeRF is provided in Figure 4.

Some studies [42,43] conducted in-depth studies on the impact of lighting or camera exposure issues on radiance fields. Among these, NeRF-W pioneered the introduction of a variable lighting model, incorporating appearance encoding into a multi-layer perceptron (MLP) for training, enabling the model to adapt to scenes under varying lighting conditions. This significantly enhanced the realism and consistency of the rendered results. However, for appearance encoding from new viewpoints, due to its inherent uncertainty, it is typically necessary to manually select reasonable values. Moreover, NeRF-W still faces limitations in handling complex lighting conditions. To address this, NeRF++ proposed an alternative solution. This approach utilizes a dual-scene representation of foreground and background to reduce the background’s interference with lighting effects, allowing for consistent rendering across different lighting conditions. Additionally, NeRF++ dynamically models the lighting, enabling a more natural simulation of changes in light source positions and intensities.

In addition to the impact of lighting, degraded image types such as splattered Gaussian noise and anisotropic blur are also significant factors that affect the rendering quality of NeRF. NeRFLiX [44] improves this situation to some extent. The model utilizes a degradation-driven viewpoint mixing method and introduces a NeRF-style Degradation Simulator (NDS), while constructing a large-scale simulated dataset, significantly enhancing flexibility. This enables the removal of NeRF artifacts and the restoration of fine details, thus greatly improving the performance of advanced NeRF models. Meanwhile, to handle motion-blurred images, Wang et al. [45] introduced a new neural radiance field called BAD-NeRF. This model uses a photometric constraint adjustment formula and averages virtual views to simulate the physical imaging process of motion blur. By continuously optimizing the difference between synthesized images and real blurred images, it accurately recovers the camera pose. Experimental results demonstrate that this method shows excellent robustness against severely motion-blurred images and inaccurate camera poses.

Unlike the improvement strategies employed by the aforementioned models in representing implicit scenes, UHDNeRF [46] have proposed an innovative novel view synthesis framework, which cleverly integrates the advantages of explicit and implicit scene representations. Within this advanced framework, implicit volumes are effectively utilized to reconstruct the low-frequency features of the entire scene, which constitute the basic structure and outlines of the scene. Meanwhile, to capture the finer details of the scene, high-frequency details are precisely captured through a sparse point cloud, ensuring the completeness and accuracy of the detailed information.

Table 1 provides a detailed overview of the significant advancements made by representative NeRF-related studies. Mip-NeRF innovatively employs conical volumes to replace traditional ray tracing methods, effectively reducing aliasing artifacts. Meanwhile, NeRF-W and NeRF++ have made important improvements in addressing lighting issues, enhancing the rendering effects under complex lighting conditions. NeRFLiX and BAD-NeRF focus on resolving artifacts caused by image degradation and motion blur, thereby improving the overall rendering quality. Additionally, UHDNeRF introduces point cloud technology into existing implicit methods to capture high-frequency details, significantly enhancing rendering quality. Although these methods have achieved breakthroughs in improving the rendering quality, they correspondingly result in increased computation time and model size. Therefore, the key challenge for researchers is to maintain efficient rendering while enhancing rendering quality.

### 3.3. Rendering Efficiency Optimization

In the field of 3D reconstruction, computational efficiency has always been a crucial research area, which is related to whether current technology can be applied in various industries. In order to improve the rendering efficiency of NeRF, researchers have proposed various innovative strategies from multiple perspectives. These strategies have shown common characteristics and development trends in many studies.

**Figure 5:**
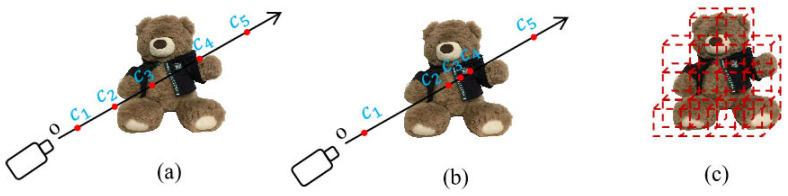
Comparison of Sampling Methods: (**a**) Uniform Sampling; (**b**) Importance Sampling; (**c**) Sparse Voxel Sampling, $c_{i}$ is the sampling point.

On the one hand, optimizing data structures and reducing redundant storage are important means to improve rendering efficiency. The neural sparse voxel field (NSVF) [47] effectively reduces the memory footprint of the model through sparse voxel division and voxel octree structure, and skips empty voxels during rendering, thus significantly improving drawing speed. These methods strive to reduce computational burden through more efficient data representation. A comparison of different sampling methods is shown in Figure 5. On this basis, researchers have also adopted multi-resolution hash coding and tensor decomposition technology to significantly improve the rendering speed (i.e., the time required to generate each frame) of NeRF. InstantNGP [48] uses multi-resolution hash coding and customized optimization strategies to store scene features in hash coding instead of relying entirely on multi-layer perceptron (MLP) weights, thus greatly improving the training speed. At the same time, TensoRF [49] uses tensor decomposition technology to represent the scene radiation field as a compact low-rank tensor, further reducing storage and computing overhead. The optimization of these methods at the algorithm level jointly promotes the improvement of rendering speed.

In addition, techniques such as recursive rendering methods, model compression, and adaptive sampling are also crucial for enhancing the rendering efficiency of NeRF. Recursive-NeRF [50] employs a recursive rendering strategy, terminating computations early when sufficient quality is achieved, thereby effectively reducing unnecessary network parameters. Zip-NeRF [51], on the other hand, optimizes feature calculations and mitigates spatial aliasing and z-aliasing phenomena through model compression and adaptive sampling techniques. These methods reduce computational load through more intelligent rendering strategies, further improving rendering efficiency.

**Figure 6:**
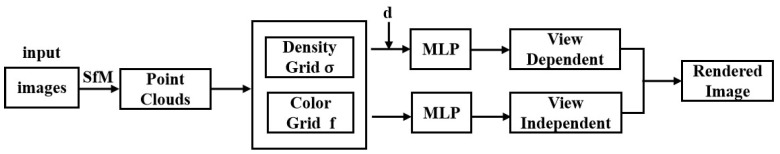
Overview of the Lightning NeRF Framework, d represents the direction of observation adapted from [53].

In other studies, researchers have focused on integrating various advanced sampling methods into the NeRF algorithm to optimize the rendering process. For example, Li et al. developed a powerful Python toolkit, NerfAcc (version v0.3.5, running on Python 3.10) [52], which provides a flexible API to integrate multiple sampling methods and optimize the rendering process through a transmittance estimator. Meanwhile, Lightning NeRF [53] combines point cloud and image-based methods, using point clouds to quickly initialize density and optimize background modeling, thereby improving processing speed and performance. These methods achieve a comprehensive improvement in rendering efficiency through the integration and optimization of multiple technical approaches. An overview of the Lightning NeRF framework is shown in Figure 6.

The above method significantly improves rendering efficiency through algorithm optimization, data structure enhancement, and the application of intelligent rendering strategies. As shown in Table 2.

### 3.4. Sparse View Synthesis

In recent years, researchers have proposed a variety of innovative methods to address the challenge of synthesizing novel views with sparse view inputs, aiming to effectively reconstruct scene details and depth information from limited view inputs. These studies not only focus on improving rendering quality but also strive to enhance the generalization ability of the model to ensure that high-quality synthetic images can be generated in different scenarios and conditions.

FreeNeRF [54] ingeniously addresses key issues in sparse sample neural rendering by introducing two main strategies: frequency regularization and occlusion regularization. These two regularization techniques not only require no additional computational resources or external supervision but also effectively suppress overfitting and mitigate visual artifacts that may arise from the density field near the camera. This significantly enhances the quality of novel view image synthesis.

Meanwhile, Seunghyeon Seo and his team have made groundbreaking advancements in the fields of 3D scene reconstruction and view synthesis with their innovative research, consecutively proposing two methods: FlipNeRF [55] and MixNeRF [56]. FlipNeRF significantly reduces floating artifacts by introducing flipped reflective rays to filter out invalid light rays and accurately reconstruct surface normals. By combining uncertainty-aware void loss and bottleneck feature consistency loss, it achieves high-quality novel view synthesis from limited perspective image inputs, greatly enhancing the accuracy and robustness of 3D scene reconstruction with few samples. On the other hand, MixNeRF further effectively learns 3D geometric structures by jointly estimating the color values’ distribution with mixed densities. It utilizes ray depth estimation to obtain geometric heights and enhances model robustness through mixed weights. This ensures more accurate scene reconstruction and higher-quality image synthesis, particularly when dealing with sparse inputs.

**Figure 7:**
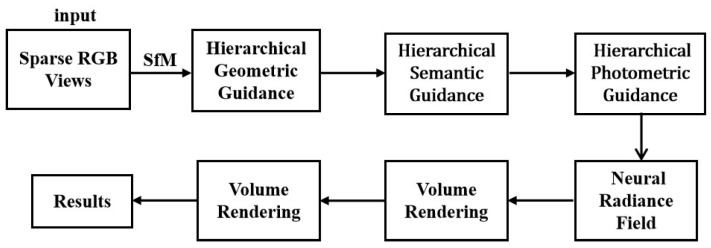
Overview of the HG3-NeRF Framework, adapted from [57].

In addition, HG3-NeRF [57] aims to enhance the consistency of geometric shape, semantic content, and appearance between different views, while addressing the performance degradation of NeRF when inputting sparse views. By combining three key techniques: hierarchical geometric guidance, hierarchical semantic guidance, and photometric guidance, it achieves higher-quality 3D scene reconstruction and view synthesis under sparse perspectives, significantly improving the model’s adaptability and robustness. An overview of the HG3-NeRF framework is shown in Figure 7.

The development of these innovative methods and technologies has not only propelled the advancement of sparse view synthesis and 3D reconstruction but also provided stronger support for future computer vision and VR applications, opening up broader possibilities for various real-world use cases.

Table 3 summarizes the evaluation metrics of the above methods on the DTU dataset, with data sourced from several relevant original papers. We evaluate the results using three metrics: Peak Signal-to-Noise Ratio (PSNR), Structural Similarity Index (SSIM), and Learned Perceptual Image Patch Similarity (LPIPS).

### 3.5. Dynamic Scene Optimization

In the field of NeRF research, dynamic scene optimization is a critical direction. The main challenge is that with the increasing complexity of real-world scenes, traditional static scene reconstruction methods can no longer meet the needs of real-time applications. Models need to have high adaptability and real-time rendering capabilities in constantly changing environments. Therefore, researchers focus on developing techniques that can effectively handle dynamic objects and their interactions to enhance the flexibility and responsiveness of models. This research not only focuses on improving rendering quality but also emphasizes maintaining consistency and stability in dynamic environments, providing stronger support for applications such as VR/AR, and robotic navigation.

Specifically, Deformable-NeRF [58] uses the multi-layer perceptron to transform spatial coordinates into regularized spatial coordinates and encodes scene states into implicit vectors, significantly improving the robustness of viewpoint synthesis in dynamic scenes. Similarly, NSFF [59] aims to reconstruct moving objects in a scene by separating the camera from dynamic objects, enabling 3D reconstruction from different viewpoints and at continuous time intervals, as well as the separation of dynamic and static parts. The D-NeRF [60] method introduces time encoding and dynamic scene decomposition techniques to successfully reconstruct and render scenes containing moving objects.

To represent dynamic scenes more deeply, NeRFPlayer [61] innovatively decomposes the neural radiance field into three types: static, deformable, and novel regions, and incorporates temporal dimension information to achieve a four-dimensional spatiotemporal neural representation. This method applies different temporal regularizations for different regions and introduces a sliding window scheme, effectively modeling the spatiotemporal field to meet the requirements of streaming playback. On the other hand, Tensor4D [62] represents dynamic scenes as 4D spatiotemporal tensors. By decomposing the 3D spatial component into a time-aware volumetric tensor, it achieves efficient memory utilization and high-quality rendering.

In addition, E-NeRF [63] leverages the characteristics of event cameras, utilizing their high dynamic range and rapid response capabilities to accurately recover the volumetric representation of scenes under complex conditions. This overcomes issues such as motion blur and inadequate lighting that are common in traditional frame-based methods, providing more precise scene information for fields such as robot navigation and augmented reality.

These innovative methods cleverly integrate advanced techniques such as multi-view learning and time-series modeling, fully utilizing temporal information to capture every subtle change in object motion. Additionally, in addressing the challenge of dynamic object modeling, researchers have delved into and adopted separation-based representation methods, effectively distinguishing and handling the complex relationships between static backgrounds and moving objects. This series of innovative measures not only significantly enhances reconstruction accuracy but also effectively reduces common issues such as dynamic blur and artifacts, opening new avenues for dynamic scene optimization and reconstruction, thus driving further development in the field.

NeRF can significantly restore the realism of a scene in 3D reconstruction, and its implementation process is simpler compared to traditional methods. However, this technology also faces several challenges and limitations. For example, lighting conditions may vary across different viewpoints, and the model may overfit to the lighting characteristics of a specific viewpoint in the training data. Moreover, it typically requires a large number of images from various viewpoints to train the model, and the training process is slow, with high demands on GPU resources; at the same time, it lacks generalization capability, as the trained model is only applicable to a specific scene and cannot be used for 3D reconstruction of other scenes; although the rendering results appear visually realistic, they resemble images from the novel viewpoints rather than the 3D models with structured features.

## 4. Three-Dimensional Reconstruction Based on 3DGS

3DGS [4] is a 3D reconstruction and rendering method based on explicit representation proposed in recent years. It achieves efficient 3D rendering by modeling the scene as Gaussian primitives, enabling significant improvements in rendering speed while maintaining image quality. Compared to traditional explicit volumetric representations (such as voxel grids), 3DGS offers a continuous and flexible geometric representation. Based on differentiable 3D Gaussian primitives, it not only enhances the descriptive power of the scene but also allows for parameterized radiance fields. This flexibility gives 3DGS a stronger adaptability when dealing with complex scenes and dynamic objects. At the same time, compared to NeRF, which relies on computationally expensive volume ray sampling, 3DGS achieves real-time rendering through tile-based rasterization, significantly improving training efficiency and avoiding unnecessary computations in empty spaces.

### 4.1. Framework of 3DGS

**Figure 8:**
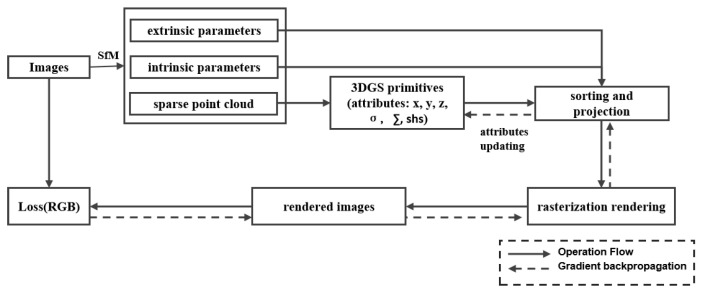
Overview of the 3DGS Framework, adapted from [4].

This method is particularly suitable for handling complex scenes and dynamic changes. Figure 8 shows an overview of the 3DGS rendering framework proposed by Kerbl. The structure of this framework consists of Gaussian initialization, densification, projection, and differentiable rasterization. 3DGS takes 3D point clouds and multi view images as inputs, initializes the 3D point clouds as Gaussian primitives with various parameters, and uses images as supervision to synthesize new perspective images through differentiable rendering.

The structure of this framework consists of Gaussian initialization, densification, projection, and micro rasterization. The rendering formula used by Kerbl et al. [4] is as follows:

(2) $$C=\sum_{i\in N}c_{i}{\sigma^{\prime }}_{i}\prod_{j=1}^{i-1}\left(1-{\sigma^{\prime }}_{j}\right)$$

where *N* is the number of Gaussians projected onto the pixel, $c_{i}$ is the color of the current Gaussian, ${\sigma^{\prime }}_{i}$ is the opacity of the current Gaussian at the pixel, and C is the rendering color of the pixel.

This innovative framework not only advances the development of 3D reconstruction technology but also provides an effective solution for modeling complex scenes. Key breakthroughs have been made in several areas of 3DGS, including enhancing real-time rendering quality, optimizing sparse-view data reconstruction, improving rendering details, and handling dynamic scene changes. Through efficient modeling and fine rendering, 3DGS enables high-precision 3D reconstruction with limited view data, while delivering high-quality rendering results in complex lighting environments and dynamic scenes. These innovations make 3DGS highly applicable in fields such as VR/AR, film production, and game development, offering strong potential for immersive experiences and real-time 3D content creation.

### 4.2. Rendering Quality Optimization

In the field of 3D graphics rendering, 3DGS has received widespread attention due to its real-time rendering capability and high-quality visual effects. However, with the increasing demand for applications, how to further optimize the rendering quality of 3DGS has become a key research direction. Optimizing rendering quality not only involves accurately capturing details and realistically reproducing lighting, but also requires effective handling of scene consistency and anti aliasing effects from different perspectives. By combining advanced technological means and algorithm innovation, researchers have made significant progress in improving the rendering quality of 3DGS.

In the process of optimizing 3DGS technology, researchers pay special attention to the application of multi-scale and multi-level strategies. For example, the MS3D method [64] employs a multi-scale 3DGS algorithm. Depending on the resolution, small-scale Gaussians are used to capture details, while large-scale Gaussians are used to maintain the overall structure of the scene. This method effectively suppresses the aliasing effect and significantly improves rendering quality and speed. At the same time, Mip-Splatting [65] combines multi-level mixing technology with Gaussian point rendering, and uses 3D smoothing filters and 2D Mip filters to significantly reduce aliasing artifacts and ensure consistent rendering effects at different resolutions. Consistency and accuracy. Together, these technologies take advantage of multi-scale processing to achieve more accurate and detailed rendering of the scene.

On the other hand, optimizing Gaussian distribution and its parameter settings is also the key to improving rendering quality. Scaffold-GS [66] dynamically optimizes Gaussian attributes by introducing anchor points, combines growth and pruning strategies, and adjusts the number and distribution of Gaussians based on their importance. This method avoids redundancy and significantly improves scene coverage and detail expression, demonstrating excellent adaptability in complex and changeable scenes. At the same time, researchers have also proposed innovative solutions to specific problems. A novel progressive propagation strategy GaussianPro [67] is proposed, which is based on MVS technology and combines the reconstructed geometric structure and patch matching technology to iteratively propagate to generate candidate pixels. In addition, this method uses photometric consistency constraints to effectively solve the problem of insufficient reconstruction accuracy in low-texture areas and significantly improve the overall reconstruction quality of 3D scenes.

In the process of improving rendering quality, GSDF [68] further advanced the technology. It combines the advantages of 3DGS and neural Signed Distance Fields (SDF), adopting a dual-branch structure consisting of the GS-branch designed specifically for rendering and the SDF-branch used for reconstruction. These two branches guide and supervise each other, complementing each other’s strengths. This not only improves reconstruction accuracy but also significantly enhances adaptability in complex scenes. Meanwhile, for super-resolution 3D scenes, SuperGS [69] proposed a method based on a two-stage coarse-to-fine training framework, which introduces Multi-Resolution Feature Gaussian Splatting and Gradient-Guided Selective Segmentation strategies to achieve efficient detail enhancement. Additionally, by leveraging pseudo high-resolution information and cross-view consistency constraints, SuperGS ensures high fidelity and consistency, significantly improving the quality and efficiency of novel view synthesis.

Table 4 summarizes the evaluation metrics of the models mentioned above on the MipNeRF 360 dataset, and the data is sourced from multiple relevant original papers (specific references can be found at the end of the article). The five metrics include Peak Signal-to-Noise Ratio (PSNR), Structural Similarity Index (SSIM), Learned Perceptual Image Patch Similarity (LPIPS), rendered frame rate (FPS), and model storage size (Memory). 

### 4.3. Rendering Efficiency Improvement

With the increasing demand for rendering complex scenes, improving the rendering efficiency of 3DGS has become a key focus of research. Currently, in the field of enhancing 3DGS rendering efficiency, researchers are primarily exploring two core directions: one is optimizing storage strategies to reduce memory usage, and the other is improving model training and rendering speed to enhance performance. Research in these two directions has not only made significant progress individually but also been intertwined to some extent, jointly driving the development of 3DGS rendering technology.

**Figure 9:**
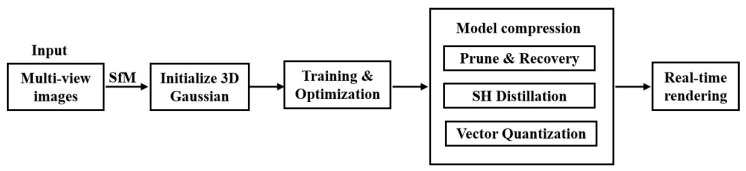
Flowchart of the LightGaussian Propagation Strategy, adapted from [70].

In terms of optimizing storage strategies, researchers have developed a series of methods to effectively reduce the memory space required for 3DGS representation. LightGaussian [70], CompGS [71] and EAGLES [72] are representative technologies in this field. The LightGaussian method achieves a compression rate of over 15 times while maintaining visual fidelity by eliminating redundant Gaussian distributions and introducing distillation and pseudo-view enhancement techniques. This approach not only significantly reduces storage requirements, but also speeds up the rendering process. The LightGaussian communication strategy flow chart is shown in Figure 9. CompGS uses a vector quantization method based on K-means to optimize Gaussian parameters and further compresses the index through run-length encoding, thereby effectively reducing storage requirements and accelerating rendering. In addition, CompGS further reduces the number of Gaussian functions by encouraging the existence of zero-opacity Gaussian functions through a regularizer. EAGLES technology introduces lightweight coding and quantized embedding methods to efficiently compress and optimize Gaussian parameters. At the same time, it uses a coarse-to-fine training strategy to accelerate the optimization process and achieves high-quality scene reconstruction on the basis of maintaining high-quality scene reconstruction. Significant reduction in storage memory and improvement in rendering speed.

In terms of improving model training and rendering speed, methods such as SuGaR [73] and Distwar [74] have shown significant results. SuGaR utilizes surface-aligned Gaussian distributions to accurately extract meshes and quickly and scalably generate meshes using a Poisson reconstruction algorithm. Additionally, SuGaR introduces an optional refinement strategy, enhancing mesh quality and rendering effects through Gaussian binding and joint optimization. This method not only improves reconstruction accuracy but also facilitates subsequent applications such as editing, sculpting, animation, and relighting. Distwar, on the other hand, is a fast and differentiable rendering technique optimized for raster-based rendering pipelines. It reduces the number of atomic operations needed by utilizing registers on the sub-cores of stream multiprocessors for thread-level reduction operations, thereby lowering the GPU workload. Furthermore, Distwar distributes atomic calculations between thread-level reduction operations on sub-cores and L2 atomic units, improving overall rendering efficiency.

Through research on the two core directions of optimizing storage strategies and improving model training and rendering speed, researchers have made significant progress in improving 3DGS rendering efficiency. These innovative methods not only reduce storage requirements, but also speed up the rendering process, providing a strong guarantee for real-time rendering of complex scenes.

### 4.4. Sparse View Reconstruction

3DGS novel view synthesis for sparse views is receiving widespread attention, which effectively captures and reconstructs the geometric and color information of complex scenes using a small number of viewpoints. Several innovative technologies in this area, such as FSGS [75], SparseGS [76], MCGS [77], and LM-Gaussian [78], although distinct in their approaches, all focus on high-quality 3D reconstruction under sparse viewpoints.

On one hand, by utilizing initialization methods such as SfM, combined with depth estimation models or depth priors, they optimize the 3D Gaussian parameters, achieving high-quality reconstruction even with limited input viewpoints. This capability not only reduces the cost and difficulty of data acquisition but also enables real-time rendering and high-quality novel view synthesis. On the other hand, these technologies adopt various strategies to improve reconstruction quality. FSGS compensates for the sparse input by using a neighbor-guided Gaussian upsampling method and incorporates depth constraints provided by a pre-trained monocular depth estimation model. SparseGS combines depth priors with generative models, explicitly constraining the optimization of 3D reconstruction and using image generation diffusion models to provide supervision for scenes with limited training set coverage. LM-Gaussian employs a robust initialization module based on visual priors to recover camera poses and generate point clouds, while introducing background-aware depth guidance and multi-modal regularized Gaussian reconstruction techniques to ensure accuracy. MCGS, in addition, generates compact and sufficient initial point sets through sparse matching and random filling strategies, enhancing the initial geometric priors, and improves the consistency of the Gaussian field through a progressive pruning strategy guided by multi-view consistency.

In addition, these technologies have achieved significant results in real-time rendering and novel view synthesis. FSGS has achieved real-time rendering speeds of over 200 FPS on multiple datasets, while also enhancing visual quality. SparseGS has enabled high-quality real-time 360° novel view synthesis. LM-Gaussian has preserved details and improved quality by iteratively applying diffusion refinement methods and video diffusion priors, making novel view images more realistic. Meanwhile, MCGS has improved model robustness, accelerated rendering speed, and reduced memory consumption by optimizing the consistency of the Gaussian field and removing low-contribution Gaussians.

### 4.5. Dynamic Scene Reconstruction

Dynamic 3D reconstruction breaks through the limitation of static reconstruction that can only capture instantaneous states by updating the model in real-time, and more realistically reflects the characteristics of the environment’s evolution over time; it utilizes multi perspective observation or time series analysis to reduce occlusion blind spots, and combines motion information to accurately distinguish between dynamic (such as pedestrians and vehicles) and static objects (such as buildings and roads), providing a foundation for scene semantic understanding; the generated real-time model has low latency and high-frequency update capabilities, directly meeting the urgent needs of dynamic environment perception in scenarios such as robot path planning, autonomous driving obstacle avoidance, and VR interaction. This is the core advantage that static reconstruction cannot achieve. Therefore, it is necessary to conduct research on dynamic 3D reconstruction based on 3DGS.

The dynamic scene reconstruction technology based on 3DGS provides a flexible and efficient method for capturing and expressing dynamic elements a number of innovative technologies, such as Deformable 3D-GS [79], V4D [80], Gaussian-Flow [81], 4D-GS [82]), and DN-4DGS [83], have demonstrated the unique advantages of 3DGS in dynamic scene reconstruction. The common feature of these technologies is that they all represent dynamic scenes as 3D Gaussian sets or dynamic 3D Gaussian particles, and use Gaussian models to capture and represent the motion details in the scene.

Specifically, these technologies optimize and refine Gaussian representations through different strategies and methods to achieve high-quality dynamic scene reconstruction. For example, Deformable 3D-GS introduces an innovative deformable 3D Gaussian representation and temporal consistency optimization, breaking through the limitations of traditional monocular dynamic scene reconstruction. This method efficiently handles complex issues such as object motion, deformation, and occlusion in dynamic environments, providing more accurate and stable dynamic 3D reconstruction. On the other hand, V4D adopts a voxel-based approach for 4D novel view synthesis. By incorporating the time dimension with spatial voxel representations, it not only improves the performance of traditional 3D models in novel view synthesis but also effectively captures and presents the details of objects changing over time, further enhancing the realism and richness of the synthesized images.

**Figure 10:**
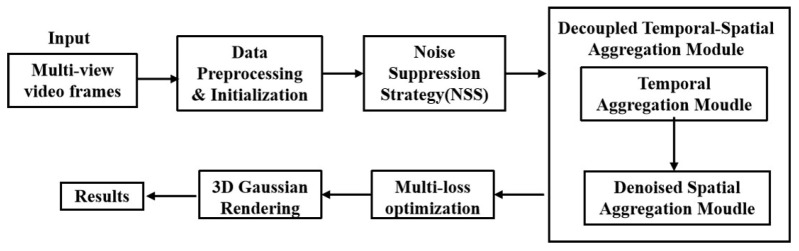
Diagram of the DN-4DGS Framework, adapted from [83].

Meanwhile, Lin proposed Gaussian-Flow, which explicitly models the temporal variation in Gaussian particle properties using a dual-domain deformation model (DDDM), enabling fast training and real-time rendering. On the other hand, 4D-GS achieves efficient high-resolution rendering through deformation fields and 4DGS technology, while introducing a decomposed neural voxel encoding algorithm to construct Gaussian features from 4D neural voxels and using a lightweight MLP to predict Gaussian deformation at new timestamps. Compared to 4D-GS, DN-4DGS significantly enhances the expressiveness of dynamic scenes by incorporating a denoising deformable network and spatiotemporal aggregation module. This ensures the purity and clarity of the image, as well as enhancing detail representation and overall coherence. The framework of DN-4DGS is shown in Figure 10.

The dynamic scene reconstruction technologies based on 3DGS have advanced the development of the field through various methods and strategies. They not only enhance the rendering quality and real-time performance of dynamic scenes but also provide new ideas and approaches for research in the fields of 3D computer vision and graphics. In the future, with continuous technological advancements and expanding application areas, 3DGS-based dynamic scene reconstruction technologies are expected to play an increasingly important role in more domains.

Table 5 summarizes the quantitative comparison of various dynamic scene reconstruction methods on the D-NeRF [60] and HyperNeRF datasets [84], with data collected from multiple original papers. The main evaluation metrics include Peak Signal-to-Noise Ratio (PSNR), Structural Similarity Index (SSIM), and Learned Perceptual Image Patch Similarity (LPIPS), with MS-SSIM used as a supplementary metric for further assessing image quality.

### 4.6. Others

In recent years, 3DGS technology has made groundbreaking progress in several cutting-edge fields, particularly in autonomous driving, large-scale scene reconstruction, Simultaneous Localization and Mapping (SLAM) technologies, and 3D editing. Its innovative applications have significantly improved performance and efficiency in these areas.

In autonomous driving scenarios, DrivingGaussian [85] technology uses static 3D Gaussian functions to sequentially and progressively model the static background, effectively capturing the structure and characteristics of the environment. At the same time, by introducing a composite dynamic Gaussian map, this technology can flexibly handle multiple moving objects, providing an accurate and efficient solution for the simulation and testing of autonomous driving. This innovation not only improves the safety and reliability of the autonomous driving system, but also lays a solid foundation for the development of future intelligent transportation systems. To address the challenges of reconstruction and rendering of large scenes, VastGaussian [86] technology achieves high-fidelity scene reconstruction by dividing multiple units and optimizing them independently. This technology utilizes block processing and parallel optimization strategies to significantly improve processing efficiency and reduce computing costs. At the same time, decoupled appearance modeling technology is introduced to effectively reduce appearance changes in rendering and further improve rendering quality. The emergence of VastGaussian technology provides strong support for real-time rendering and interaction of large scenes.

In the field of SLAM technology, IG-SLAM [87] and GS-SLAM [88,89] are two important technical branches, showing different application prospects of 3DGS technology. IG-SLAM uses dense SLAM methods for tracking, combined with 3D Gaussian point rendering technology, and optimizes maps through deep uncertainty to achieve fast and realistic 3D reconstruction. GS-SLAM combines the advantages of NeRF and SLAM, uses 3D Gaussian to represent scenes, and achieves ultra-fast rendering speed and high-quality mapping through splatting-based rasterization technology. Both technologies are promoting the development of SLAM technology, providing new solutions for real-time 3D reconstruction and positioning, and showing broad application potential in fields such as autonomous driving and VR.

In addition, in terms of 3D editing, the introduction of PixelSplat [88] technology provides users with a more flexible and efficient 3D creation tool. This technology leverages a feedforward model to reconstruct a 3D radiance field parameterized by 3D Gaussian primitives from input image pairs, achieving scalable training and fast 3D reconstruction. At the same time, the generated 3D radiation field is interpretable and editable, providing users with rich creative materials and convenient means of creation in multiple fields such as VR/AR, game development, and architectural design. The emergence of PixelSplat technology has further promoted the development of 3D editing technology, bringing users a richer creative experience and a broader creative space.

## 5. Challenges and Prospects

The 3D reconstruction technology has undergone significant technological evolution from traditional multi view geometry methods, to NeRF based on neural implicit representation, and to 3DGS with efficient and real-time rendering. Each technological iteration not only brings significant improvements in modeling efficiency and reconstruction quality but also promotes the development of 3D vision systems from static analysis to dynamic, real-time, and editable directions.

Traditional methods such as SfM and MVS, represented by classic algorithms, mainly rely on dense multi view image inputs to restore 3D structures through geometric matching. This type of modeling method, with Mesh as the core, has high geometric accuracy and is widely used in engineering fields such as architectural surveying and cultural heritage protection. However, traditional methods also suffer from poor adaptability to dynamic scenes, poor reconstruction quality of weak texture or complex structural areas, sensitivity to lighting changes, and cumbersome model modification and editing processes. Despite mature technology and sophisticated tools, traditional Mesh reconstruction methods often struggle to provide the required rendering speed and dynamic update capability in application scenarios that require high real-time and flexibility, such as autonomous driving and digital twins. NeRF uses differentiable rendering technology to learn the color and density of each spatial position in the scene from limited static images, achieving extremely realistic volume rendering effects, especially in soft shadows, global lighting, and transparent material modeling. However, NeRF still faces many challenges: slow rendering speed and difficulty in meeting real-time interaction requirements; limited support for dynamic scenes and susceptibility to interference from moving objects during training; implicit representation is difficult to directly edit; at the same time, the training cost is high, and there are strict requirements for computing power and data quality. These factors limit its widespread implementation in engineering practice. In contrast, 3DGS represents the scene as Gaussian elements with parameters such as opacity, color and covariance, and spherical harmonics, and synthesizes images from a new perspective through rasterization rendering, significantly improving rendering efficiency while maintaining rendering quality. 3DGS supports real-time modeling and rendering of dynamic scenes, with good lighting adaptability and editability. It can achieve high frame rate rendering under moderate hardware conditions, greatly expanding its applicability in real-time application scenarios such as AR/VR, autonomous driving, and digital twins. In addition, the 3DGS model structure is open and easy to integrate with existing tools, supporting direct editing of Gaussian parameters, significantly reducing post production and iteration costs. However, 3DGS still has certain limitations. For example, in large-scale scenarios, the number of Gaussians is large and requires high storage and memory resources. The accuracy of the original 3DGS model that relies on camera pose is also limited by the accuracy of SfM calculations. These factors may pose challenges in high-precision and high dynamic scene modeling. Future research should focus on enhancing the robustness of 3D reconstruction technology, reducing computational complexity, and expanding its applicability to address challenges in dynamic scenes, fast feedback, and computational efficiency. Specifically:

1. NeRF and 3DGS face high requirements for input data quality and quantity, as well as a heavy reliance on specific technologies such as SfM. Some studies [90,91,92] use multimodal data fusion to obtain high-quality initial data in order to improve the robustness and generalization of reconstruction, and reduce dependence on external tools. This is also an important direction for future research.
2. Reducing computational complexity and enhancing processing speed: Although NeRF and 3DGS have proposed numerous methods to improve efficiency, the computational complexity and resource consumption associated with their corresponding applications remain a non-negligible issue. Future research needs to explore more efficient network structures and algorithms to reduce computational complexity and increase processing speed, thereby making them more suitable for practical application scenarios.
3. Expanding generalization capability and adapting to diverse scenarios: 3D reconstruction technology has been widely applied in fields such as VR/AR and intelligent transportation systems. In the future, with the continuous expansion of application scenarios, such as deeper applications in autonomous driving and robot navigation, higher demands will be placed on the technology’s ability to generalize.
4. Integrating advanced technologies to form comprehensive solutions: By combining advanced technologies such as deep learning, computer vision, and sensor technologies with 3D reconstruction techniques, more comprehensive and powerful solutions can be developed. For example, by further integrating generative diffusion models [93,94] with 3DGS technology, more accurate and real-time 3D reconstruction can be achieved. Additionally, the new model DepthSplat [95] combines 3DGS with multi-view depth estimation, improving performance in occlusion and complex texture scenarios.

## 6. Conclusions

This article reviews the development history of 3D reconstruction technology, from traditional methods to NeRF and 3DGS based on neural networks. Traditional methods such as SfM and MVS have been widely applied in engineering fields like architectural surveying and natural resource management. However, when dealing with dynamic scenes, weak texture areas or complex structures, they have poor adaptability to changes in lighting, and model editing is difficult to meet the requirements of real-time performance and flexibility. NeRF’s implicit reconstruction can generate high-quality results, especially excelling in soft shadow and transparent material modeling. However, its high computational cost and slow training and rendering speeds limit its real-time interactive applications in engineering practice. In contrast, 3DGS’s explicit reconstruction achieves a better balance between image quality and rendering efficiency, and demonstrates advantages in real-time rendering and editable performance. However, it still faces challenges in terms of memory usage in large-scale scenarios, stability in dynamic scenarios, and cross-scenario knowledge transfer. To sum up, although 3D reconstruction technology has made significant progress in modeling accuracy, visual realism and rendering performance, it still faces many bottlenecks. Future research should focus on breaking through the scalability of large-scale outdoor environments, robustness in dynamic and unconstrained scenarios, as well as computational efficiency in real-time applications. Meanwhile, the deep integration of 3D reconstruction with fields such as AR/VR, robotics and autonomous driving will bring broader prospects for its practical application. With the continuous cross-development of artificial intelligence and computer graphics, 3D reconstruction is expected to become an important driving force for cross-domain digital transformation and the evolution of intelligent systems.

## Figures and Tables

**Table 1:** Technical Innovations and Optimization Results of NeRF Rendering Quality Optimization Algorithms.

Optimization Method	Technological Innovation	Optimization Results
Mip-NeRF [41]	Introducing a new integrated encoding and replacing ray tracing with cone tracing	Reduces image aliasing and jagged artifacts
NeRF-W [42]	Introduced a variable lighting model and used latent variables to represent appearance features under different viewpoints and conditions	Adapted to varying lighting conditions, generated diverse views, improved image quality and robustness, and enhanced training efficiency
NeRF++ [43]	Utilized a dual-scene representation for foreground and background, and dynamically modeled the lighting conditions	Enhanced scene details and lighting consistency, achieving higher-quality multi-view reconstruction
NeRFLiX [44]	A degradation-driven view blending method is used, along with the introduction of a NeRF-style Degradation Simulator (NDS)	Effectively restores degraded rendered composite frames, reduces image artifacts, and improves rendering quality
BAD-NeRF [45]	Introduces a photometric constraint adjustment formula to simulate the physical imaging process of motion blur	Effectively reduces motion blur in images and enhances rendering quality
UHDNeRF [46]	Combines the advantages of explicit and implicit scenes, using sparse point clouds to capture high-frequency details	Extracts rich texture details and has an advantage in rendering results at extremely high resolutions

**Table 2:** Technical Innovations and Optimization Results of NeRF Rendering Efficiency Optimization Algorithms.

Optimization Method	Technological Innovation	Optimization Results
NSVF [47]	Sparse voxel partitioning and voxel octree structure are employed, along with the implementation of an efficient ray tracing algorithm	Significantly reduced memory consumption, achieved a more than 10× speedup in inference, and produced higher-quality results
InstantNGP [48]	Introduced a multi-resolution hashing encoding method, combined with CUDA, to fully leverage the parallel computing power of GPUs	Reduced memory consumption, compressing the training time of NeRF from hours to minutes or even seconds
TensoRF [49]	Proposed a vector-matrix decomposition method to decompose the scene into compact low-rank tensors	Reduced the memory required for rendering, while significantly decreasing the training time of NeRF
Recursive-NeRF [50]	Adopted a recursive rendering strategy by processing scene information in layers, while also proposing an innovative multi-stage dynamic growth method	Reduced unnecessary network parameters, resulting in more efficient synthesis while improving rendering quality
Zip-NeRF [51]	Introduced efficient data compression techniques while employing multi-level feature representations	Reduced memory consumption while optimizing computational efficiency and preserving details
NerfAcc [52]	Combined multi-sampling methods and optimized the rendering process through a transmissivity estimator	Rendering speed improved by 1.5 to 20 times, while rendering quality was enhanced
Lightning NeRF [53]	Combining point clouds and images, using point clouds to quickly initialize density and optimize background modeling	Training speed increased by 5 times, rendering speed improved by 10 times, and view synthesis quality significantly enhanced

**Table 3:** Comparison of related methods for sparse view synthesis on the DTU dataset. Arrows denote the direction of improvement (**↑** higher is better; **↓** lower is better), and underlined values indicate the best results.

Methods	PSNR ↑			SSIM ↑			LPIPS ↓		
	3-View	6-View	9-View	3-View	6-View	9-View	3-View	6-View	9-View
PixelNeRF [39]	16.82	19.11	20.40	0.695	0.745	0.768	0.270	0.232	0.220
FreeNeRF [54]	19.92	23.25	25.38	0.787	0.844	0.888	-	-	-
FlipNeRF [55]	19.55	22.45	25.12	0.767	0.839	0.882	0.180	0.098	0.062
MixNeRF [56]	18.95	22.30	25.03	0.744	0.835	0.879	0.203	0.102	0.065
HG3-NeRF [57]	19.37	23.35	25.87	0.759	0.855	0.891	0.177	0.094	0.061

**Table 4:** Quantitative Comparison Results of 3DGS Improvement Solutions on the MipNeRF 360 Dataset. Arrows denote the direction of improvement (**↑** higher is better; **↓** lower is better), and underlined values indicate the best results.

Methods	Year	PSNR ↑	SSIM ↑	LPIPS ↓	FPS ↑	Memory ↓
3DGS [4]	2023	27.21	0.815	0.214	134	734
GSDF [68]	2024	29.38	0.865	0.185	-	-
Mip-Splatting [65]	2024	27.79	0. 827	0.203	-	-
Scaffold-GS [66]	2024	28.84	0.848	0.220	102	156
GaussianPro [67]	2024	27.92	0.825	0.208	108	-
SuperGS [69]	2024	29.44	0.865	0.130	47	123

**Table 5:** Quantitative Comparison on D-NeRF and HyperNeRF.Arrows denote the direction of improvement (**↑** higher is better; **↓** lower is better), and underlined values indicate the best results.

Methods	D-NeRF			HyperNeRF	
	PSNR ↑	SSIM ↑	LPIPS ↓	PSNR ↑	MS-SSIM ↑
Gaussian-Flow [81]	34.27	0.98	0.03	26.3	0.862
Deforable 3D-GS [79]	39.51	0.99	0.01	-	-
V4D [80]	33.72	0.98	0.02	24.80	0.832
4DGS [82]	34.05	0.98	0.02	25.20	0.845
DN-4DGS [83]	-	-	-	25.59	0.863

## Data Availability

Data sharing is not applicable to this article as no new data were created or analyzed in this study.
